# The comparative study of resonance disorders for Vietnamese and Korean cleft palate speakers using nasometer

**DOI:** 10.1186/s40902-017-0108-2

**Published:** 2017-04-25

**Authors:** Yu-Jeong Shin, Yongsoo Kim, Hyun-Gi Kim

**Affiliations:** 1 Department of Speech-Language Therapy, Howon University, Gunsan City, Republic of Korea; 2 Department of Oral & Maxillofacial Surgery, Institute of Oral Bioscience, School of Dentistry, Clinical Research Institute of Chonbuk National University Hospital, Chonju City, Korea; 3 Department of Speech-Language Therapy, Graduate School, Research Institute of Speech Sciences, Chonbuk National University, 20 Gungiro Road, Duckjin-Gu, Jeonju City, Chonbuk 561-180 Korea

**Keywords:** Nasalance, Ethnic difference, Nasometer, Cleft palate

## Abstract

**Background:**

Nasalance is used to evaluate the velopharyngeal incompetence in clinical diagnoses using a nasometer. The aim of this study is to find the nasalance differences between Vietnamese cleft palate children and Korean cleft palate children by measuring the nasalance of five oral vowels.

**Methods:**

Ten Vietnamese cleft palate children after surgery, three Vietnamese children for the control group, and ten Korean cleft palate children after surgery with the same age participated in this experimentation. Instead of Korean control, the standard value of Korean version of the simplified nasometric assessment procedures (kSNAP) was used.

**Result:**

The results are as follows: (1) the highest nasalance score among the Vietnamese normal vowels is the low vowel /a/; however, that of Korean normal vowels is the high vowel /i/. (2) The average nasalance score of Korean cleft palate vowels is 18% higher than that of Vietnamese cleft palate vowels. There was a nasalance score of over 45% among the vowels /e/ and /i/ in Vietnamese cleft palate patients and /i/, /o/, and /u/ in Korean cleft palate patients.

**Conclusion:**

These different nasalance scores of the same vowels seem to cause an ethnic difference between Vietnamese and Korean cleft palate children.

## Background

Cleft palate speakers generally have a deviation in speech resonance because of velopharyngeal incompetence (VPI). Traditionally, evaluation of resonance disorder was mostly subjective evaluation which classifies degree of hypernasality such as stage 4 or stage 5. However, it was difficult for speech therapists who have difficulties with encountering cleft palate speakers to evaluate the degree of hypernasality. It was also hard to apply clinical experiments because the results of speech evaluations differ from each therapist.

Nasometer is the equipment designed to solve the problems mentioned above, and it can measure nasal sounds. It is a computer-based instrument with an attached hardware sound card. If an acoustic sound signal from outside has a value of 100%, each acoustic energy from the oral and nasal cavity has a value of 50%. Acoustic energy is measured by the dynamic microphone which is attached to a partitioned wall between the nose and the mouth, and it can be represented by a nasalance score.

Recently, the differences between nasalance score of gender, age, dialect, and ethnic group are shown by using a nasometer in speech researches, which was mainly used in clinical experiment. Seaver claimed that female speakers were found to have higher nasalance score than male speakers by the study of nasalance score difference between English speaker genders [1]. In French speakers, female speakers have higher nasalance score than male speakers as well [2]. However, Pranthanee’s study of 13-year-old normal children in Thailand and Kavanaugh’s study of normal children in Australia reported that there is no significant difference between nasalance score by gender [3, 4].

According to Warren, no differences were found in nasalance score depending on age groups [5]. On the other hand, Alan claimed that adults have lower nasalance score than that of children, and this is because children have lack of stability compared by adults when they speak, and they do not make a velopharyngeal control according to the length of the sounds [6].

In the research of ethnic groups, the result of nasalance score between an American who speaks standard American English and an African American who speaks Mid-Atlantic dialect reported that standard American English speakers were found to have much higher nasalance score [7]. In sum, most researchers examined nasalance score by using nasometer depending on gender and dialect to Caucasians, but Warren suggests that further study about facial shape or shape of nose by different ethnic groups is needed because the shape of airway affects listener [8].

Nasalance score variation in Japanese speakers of Mid-west Japanese dialect proposed only criteria to evaluate nasal sound, not the difference depending on regional dialect [9]. The result of difference between the dialect of Mid-Atlantic province and Ontario of Canada regional dialect reported difference of nasalance score depending on regional differences [3]. In terms of language differences, according to Fletcher, Spanish children have lower nasalance score than English children [10, 11]. There is a difference of nasalance score between a dialect from an English-speaking region and French-speaking area in Canada [12]. Van Doorn reported that Canadian children have lower nasalance score, while American children have higher score than Australian children from the comparison of nasalance score using “Zoo passage” between Australian and North American children. That is, nasalance score has been shown to depend on the difference of languages [13].

So far, the various subjects of measuring nasalance by objective method are insufficient, especially comparison studies of nasalance depending on age group and language. Moreover, the study of nasalance between Westerners and Asians is quite restricted. The aim of this study is to investigate the nasalance differences between Vietnamese cleft palate children and Korean cleft palate children and to provide a database for standard evaluation of nasal disorder of Vietnamese of VPI, also to gather evaluation database of hypernasality for international standardization in terms of anthropological linguistics and comparative linguistics.

## Methods

### Subjects

Ten Vietnamese cleft palate children after surgery, three Vietnamese children for control group, and ten Korean cleft palate children after surgery with the same age participated in this study. Standard kSNAP values are applied for the Korean control group (Table 1).

**Table 1:** Subjects of the study

Subject	Cleft palate children after surgery	Control group
Vietnamese	10	3
Korean	10	kSNAP

### Speech samples

The measuring sounds included five similar vowels from the Vietnamese and Korean vowel system (Table 2). Five vowels from the Vietnamese which has a corresponding property with the Korean were read by a Vietnamese interpreter. The subjects repeated speech samples of what the interpreter read.

**Table 2:** Stimulus material for measuring nasalance

	Speech samples
Vietnamese vowels	/âm, di, t'u, lê, cô, /
Korean vowels	/a, i, u, e, o/

### Methods

**Fig. 1:**
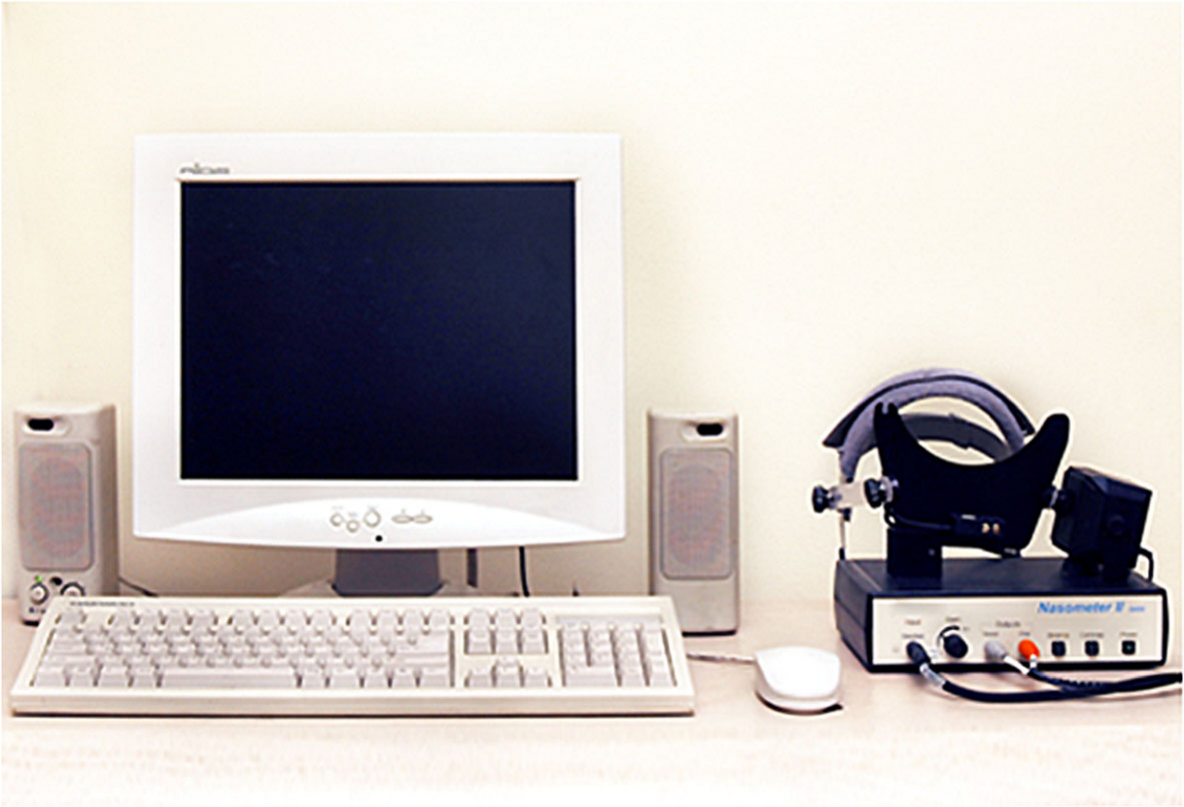
Nasometer II, Model 6400 (KayPentax, USA, 2003)

Nasometer II, Model 6400 (KayPentax, USA, 2003) connected to a laptop was used to measure the nasalance score (Fig. 1).

### Procedures

The experiment has been conducted in the dental school in Hue University in which a volunteer team led by Shin Hyo-keun from the Department of Oral & Maxillofacial Surgery, College of Dentistry, Chonbuk National University, and also it was relatively quiet. The subjects and the interpreter presented. The subjects had the cleft palate surgical operation from Hue University already. Once the interpreter read a Vietnamese vowel, the subject inputs data after repeating what the interpreter said following instruction from the interpreter. The analysis was conducted after choosing the most accurate utterance among the reading of each stimulus material three times repeatedly by the subject. Aside from the nasometer, the experiment was also carried out with a video recording in order to seek accuracy of utterance. All investigators carried out the research within legal and ethical obligation of the Helsinki declaration. Informed consent was obtained from all parents/guardians of the children for collecting data which does not contain personal identifiable information for publication.

## Results and discussion

The statistical comparisons of the nasalance score data between Korean and Vietnamese cleft palate children with comparison group are displayed in Table 3. Results show that there are two hypernasality and one hyponasality in Vietnamese cleft palate children. Comparing with the control group, hyponasality was presented almost in every vowel except in vowel /a/. The average value of nasalance score (9.6%) was lower than that of the control group (11.6%). The front vowel /e/ and /i/ have high nasalance in Vietnamese hypernasality children (/e/, 54.5%; /i/, 45.5%), while vowel /i/ has the highest nasalance score following vowel /o/ and /u/ (more than 55%) in Korean cleft palate children. In the nasalance difference between Vietnamese cleft palate children and Korean cleft palate children, nasalance score of vowel /e/ from Vietnamese cleft palate children has higher score than that from Korean children but Vietnamese cleft palate children have lower nasalance score than Korean cleft palate children in the rest of the vowels. Nasalance score between the Vietnamese control group and Korean control group has nearly the same score, but Vietnamese hypernasality cleft palate children obtained slightly lower nasalance score than the Korean hypernasality cleft palate children.

**Table 3:** Nasalance for Vietnamese and Korean cleft palate children with the control group (%)

Subjects	/a/	/e/	/i/	/o/	/u/	Total
Viet. hyponasality	19.0	7.0	9.0	6.0	7.0	9.6
Viet. hypernasality	22.5	54.5	45.5	28	23	34.7
Kor. hypernasality	31.4	43.7	76.9	55.4	57.9	53.0
Viet. control group	19.0	8.0	12.0	11.0	8.0	11.6
Kor. control group	8.6	8.7	22.3	8.4	10.0	11.6

Nasogram shows the slope according to the opening and closing degree of the uvula. The negative slope of the nasogram presents the closing process of the uvula, while the positive slope shows the opening process of the uvula. There is a marked contrast between Vietnamese hypernasality and hyponasality cleft palate children from the average value of slope (Table 4). Hypernasality cleft palate children show five times steeper increase in slope than hyponasality cleft palate children. This result is because there is no change in slope for hyponasality cleft palate children except vowel /a/.

**Table 4:** Degrees of the slopes represented in Vietnamese hyponasality and hypernasality

Subject	/a/	/e/	/i/	/o/	/u/	Total
Viet. hyponasality	26.8	2.0	2.4	0.0	4.4	7.12
Viet. hypernasality	24.0	58.0	39.0	31.0	20.0	34.4

**Fig. 2:**
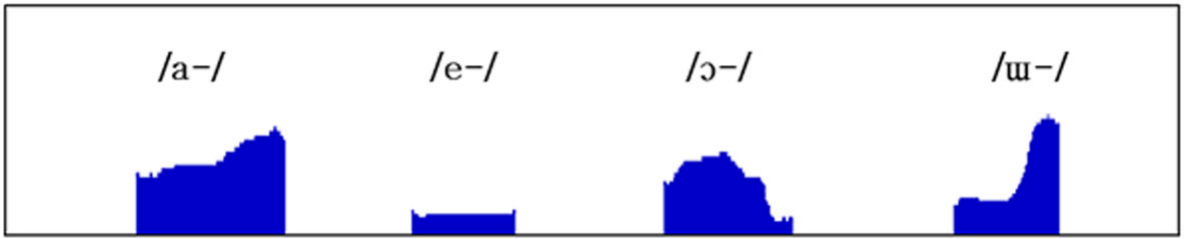
Nasogram slope types of Vietnamese cleft palate children

Nasogram of Vietnamese cleft palate children has four types as presented in Fig. 2. Vowel /a-/ shows a gradually increasing curve as 14.1–17.2%, and /e-/ presents a steady curve with no significant change, /ɔ/ shows a steep rise, steady curve, and steep descent as 62.1–9.6–66.6%, and /ɯ/ shows a steady rise following a steep increase as 5.04–46.6%.

The study of nasalance score based on the language, gender, age, group, and regional dialect has been presented in clinical experiments for resonance disorder thanks to commercialized nasometer which is designed to measure by hyper and hyponasalance score. To sum up the previous study, this has been broadly applied not only in anthropological linguistics which has a difference in nasalance score depending on oral cavity but also in comparative linguistics which is a study of comparing nasalance scores between each language.

According to literature, the study of nasalance was carried out by clinicians, rather than linguists, who tried to solve the resonance disorder caused by velopharyngeal insufficiency by surgery, but because of so many cases of communication disorder caused by hypernasality after surgery, clinicians have been interested in speech management. The nasometer was developed because an objective hypernasality evaluation was demanded rather than any other subjective evaluation. Also, there was a problem of reliability of evaluation result, since inspectors such as clinicians and language therapists can make errors, no matter how they are well-trained. Some drawbacks were pointed out that the study of nasalance score should be conducted according to the property of language, gender, age difference along with oral and nasal cavity [12].

The study of nasalance that classified each language was carried by the comparison between Spanish and English, Canadian English and French, and Australian English, American English, and Canadian English. Comparison of the nasalance between Korean and Vietnamese speakers, as a Latin language family, was not yet introduced. This study evaluates the difference of nasalance depending on ethnic groups and language groups, based on the stimulus material which is standardized by a dental school in Chonbuk National University, which compared cleft palate patients in Hue, Vietnamese with the control group and Korean. This is also the basis of experiment for cleft palate patients with resonance disorder. Five Korean vowels /a, i, u, e, o/ and equivalent Vietnamese vowel sounds were selected and analyzed.

There is no significant difference from nasalance in the comparison between the Vietnamese and Korean control group. /a/ has the highest nasalance in the Vietnamese control group while /i/ has the highest nasalance score in the Korean control group. Korean hypernasality cleft palate children have 18% higher than Vietnamese from comparison of nasalance score. There is an assumption that Vietnamese cleft palate children are more ethnically effectual for the surgery because Vietnamese subjects are at the early stage of verbal development despite the same surgery. Especially, the nasalance score of Vietnamese hyponasality cleft palate child, which was low except in the vowel /a/, relied on the difference in ethnic group.

**Fig. 3:**
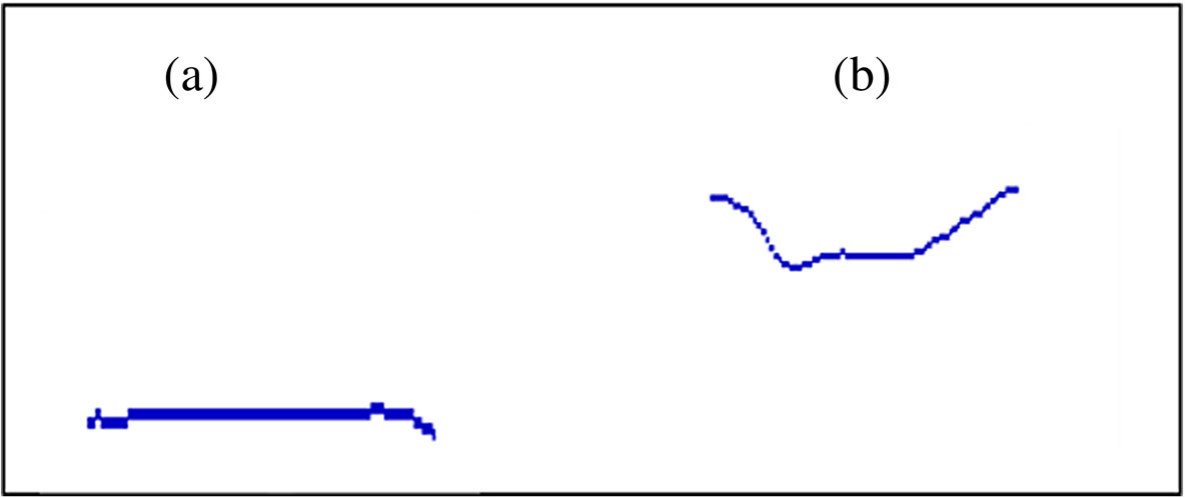
Comparison of the nasalance slope between Vietnamese hyponasality (**a**) and hypernasality (**b**) cleft palate children

If the boundary value of Korean hypernasality is 45% (Kwon and Shin, 1994), Vietnamese cleft palate children have high nasalance score in /e/ and /i/, while Koreans have high nasalance score in /i/, /o/, and /u/. Vietnamese hyponasality cleft palate children have very low degree of nasalance (between 6 and 9%) except in /a/. Nasometer between hypernasality and hyponasality cleft palate children shows remarkable differences. In comparison nasogram, the degree of slope between hypernasality and hyponasality Vietnamese cleft palate children is almost parallel (Fig. 3a), while nasogram of hypernasality cleft palate children shows a steep descent-parallel-steep rising (Fig. 3b). Also in the comparison of every vowel, Vietnamese hypernasality cleft palate children have the nasogram which is five times steeper than hyponasality children have. Based on these results, it can be assumed that the characteristic of nasalance may vary depending not only on ethnic or language groups but also on the result of surgical operations.

## Conclusions

This study is concerned about the analytic comparison of nasalance scores between Vietnamese and Korean cleft palate children with the control group in terms of ethnic and language differences. There are some arguments what we obtained from the study.

1. According to the comparison of nasalance scores, depending on the ethnic group, Vietnamese have the highest nasalance score in vowel /a/ while Koreans have the highest score in vowel /i/. Vietnamese vowels /a/ and /o/ have higher nasalance score than Korean vowels in accordance with the comparison of nasalance scores by language difference.
2. Vietnamese hypernasality cleft palate children had 18% lower score in average value of nasalance score than Korean hypernasality cleft palate children. Hearing diagnosis of hypernasality exceeded the standard value of nasalance score (45%) in Vietnamese hypernasality cleft palate children in /e/ and /i/, while Korean hypernasality cleft palate children was presented higher value (more than 45%). This result suggests that stimulus questions which are suitable for different languages are required because the evaluation differs from ethnic or language groups. Especially that the nasalance score of Vietnamese cleft palate children has the higher score in the vowel /e/ while /i/ has the highest score in Korean. The difference between maximum values of hypernasality is 22.4%.
3. The average nasalance score of Vietnamese hypernasality cleft palate children shows five times higher than Korean, and hyponasalance score was low in every vowel except /a/. There is a remarkable distinction between hyponasality cleft palate children and hypernasality cleft palate children. The former shows low and lineal nasogram scope, while the latter shows high steep falling-parallel-steep rising curve in a sequence.

## Abbreviations

kSNAP-test: Korean version of the simplified nasometric assessment procedures test
VPI: Velopharyngeal incompetence; velopharyngeal insufficiency
